# When Carcinoembryonic Antigen Misleads: A Benign Cause of Tumor Marker Elevation

**DOI:** 10.7759/cureus.89341

**Published:** 2025-08-04

**Authors:** Samer Alhasan, Azeez Alzafiri, Hisham Tharwat, Ramia Alhasan, Nadia Alrashidi

**Affiliations:** 1 Internal Medicine, New Jahra Hospital, Al Jahra, KWT; 2 Internal Medicine, Al Jahra Hospital, Al Jahra, KWT

**Keywords:** anxiety, carcinoembryonic antigen, carcinoembryonic antigen (cea), cea, cholecystitis, diagnostic cascade, health screening, tumor marker

## Abstract

Carcinoembryonic antigen (CEA) is a commonly used tumor marker, primarily for the surveillance of colorectal and other gastrointestinal malignancies. However, its diagnostic specificity is limited, as CEA levels may be elevated in several benign conditions. This case report aims to highlight the potential diagnostic confusion and psychological distress caused by incidental CEA elevation in asymptomatic individuals when tested outside of an appropriate clinical context. We describe the case of a 39-year-old Kuwaiti woman who underwent a routine health check-up at a private laboratory, during which CEA was measured despite the absence of cancer-related symptoms. Her CEA level was markedly elevated, prompting anxiety and a cascade of investigations. Clinical history, physical examination, laboratory testing, endoscopic procedures, and imaging were employed to determine the cause of elevation. The patient's elevated CEA level (49.02 ng/mL) was not associated with any detectable malignancy. Her past medical history included a laparoscopic sleeve gastrectomy a year earlier. Clinical assessment revealed chronic dyspepsia, anemia, and right upper quadrant tenderness. A comprehensive workup, including colonoscopy, upper gastrointestinal (GI) endoscopy, and contrast-enhanced CT imaging excluded neoplastic processes. Abdominal ultrasound revealed chronic calculous cholecystitis. Following laparoscopic cholecystectomy, her symptoms improved and repeat CEA levels normalized, confirming a benign inflammatory etiology. This case illustrates the limitations of CEA as a screening tool and the importance of interpreting tumor markers within the appropriate clinical context. Routine ordering of tumor markers in asymptomatic patients can result in unnecessary anxiety, diagnostic delays, and resource use. Clinicians should rely on symptom-guided evaluations rather than indiscriminate testing.

## Introduction

Carcinoembryonic antigen (CEA) is a glycoprotein that functions as a tumor-associated marker, most commonly used in the surveillance of colorectal cancer. It has clinical utility in monitoring disease recurrence or response to therapy in selected malignancies, including colorectal, gastric, pancreatic, and lung cancers [1,2]. Despite its widespread use, CEA lacks diagnostic specificity. Elevated levels may also be observed in a variety of benign conditions such as inflammatory bowel disease, chronic obstructive pulmonary disease [3-5], liver dysfunction, and biliary tract disorders such as chronic cholecystitis [6,7], as well as in smokers and individuals taking certain medications. The increasing availability of commercial health screening packages has led to the inclusion of tumor markers, including CEA, in asymptomatic individuals without clinical justification. This practice can result in incidental findings that provoke anxiety, lead to diagnostic cascades, and strain healthcare resources [6,8]. Current guidelines do not recommend the use of CEA for cancer screening in asymptomatic populations due to its low positive predictive value in this setting [2,9]. Instead, evidence-based colorectal cancer screening strategies, such as fecal immunochemical testing (FIT), fecal occult blood testing (FOBT), multi-target stool DNA testing (mt-sDNA), and CT colonography, are recommended starting at age 45-50, depending on the risk level [10,11]. Moreover, the interpretation of elevated tumor markers is further complicated when patients have comorbid conditions that may independently influence these values. Post-bariatric surgical changes, nutritional deficiencies, or chronic inflammation may contribute to nonspecific symptomatology and confound diagnostic reasoning. This case report presents a 39-year-old woman who was found to have a markedly elevated CEA level during a routine check-up, leading to extensive investigations and psychological distress. The diagnostic workup eventually identified chronic calculous cholecystitis as the most likely cause. The aim of this report is to emphasize the importance of context-driven interpretation of tumor markers, and to caution against the indiscriminate use of such tests in routine health screenings.

## Case presentation

A 39-year-old Kuwaiti woman was referred to our internal medicine clinic in January 2024, following a routine health screening conducted at a private diagnostic center. The check-up, which included tumor marker testing despite the absence of clinical indication, revealed a markedly elevated CEA level of 45.89 ng/mL (reference range: 0-3.2 ng/mL). This unexpected result caused significant psychological distress and concern about an underlying malignancy. The patient reported a history of chronic dyspepsia, generalized fatigue, and a 25 kg weight loss over the preceding year. Importantly, she had undergone laparoscopic sleeve gastrectomy approximately a year earlier, which she identified as the primary reason for her weight loss. She denied changes in bowel habits, melena, hematochezia, night sweats, or appetite loss. Her family history was notable for a father who had died of colorectal cancer at the age of 65, a year prior. Physical examination revealed pallor and mild tenderness in the right upper quadrant. No lymphadenopathy, hepatosplenomegaly, or abdominal masses were noted.

Laboratory investigations demonstrated microcytic hypochromic anemia, with a hemoglobin level of 10.5 g/dL, mean corpuscular volume (MCV) of 72 fL, and mean corpuscular hemoglobin (MCH) of 23 pg. Iron studies revealed low serum iron (4.8 µmol/L) and ferritin (5.7 ng/mL), along with vitamin B12 deficiency (133 pg/mL). A repeat CEA test performed at our hospital confirmed a further elevation to 49.02 ng/mL. Inflammatory markers were within normal range, including white blood cell count (WBC: 7.9 × 10⁹/L), procalcitonin (PCT: 0.01 ng/mL), and C-reactive protein (CRP: 3 mg/L) (Table 1).

**Table 1:** Hematological and biochemical investigations

Parameter	Result	Reference range
Hemoglobin (Hb; g/dL)	10.5	12.0-16.0
Mean Corpuscular Volume (MCV; fL)	72	80-96
Mean Corpuscular Hemoglobin (MCH; pg)	23	27-33
White Blood Cell Count (WBC; × 10⁹/L)	7.9	4.0-11.0
C-Reactive Protein (CRP; mg/L	3	<5
Procalcitonin (PCT; ng/mL)	0.01	<0.05
Serum iron (µmol/L)	4.8	10-30
Ferritin (ng/mL)	5.7	15-150
Vitamin B12 (pg/mL)	133	180-914
CEA (initial; ng/mL)	45.89	0-3.2
CEA (repeat; ng/mL)	49.02	0-3.2

Liver function tests, including bilirubin, aspartate aminotransferase (AST), alanine aminotransferase (ALT), alkaline phosphatase, and gamma-glutamyl transferase (GGT), were also normal, excluding hepatic dysfunction as a contributing factor. Given the combination of anemia, reported weight loss (interpreted as likely intentional given her history of bariatric surgery), positive family history of colorectal cancer, and elevated CEA, a thorough diagnostic work-up was initiated. Stool occult blood testing was performed on three separate occasions and was negative.

Abdominal ultrasonography identified gallstones and thickening of the gallbladder wall, consistent with chronic calculous cholecystitis. A contrast-enhanced computed tomography (CT) scan of the chest, abdomen, and pelvis revealed no suspicious lesions or lymphadenopathy. Upper gastrointestinal (GI) endoscopy revealed mild gastritis, and a rapid urease (CLO) test was positive for Helicobacter pylori. Colonoscopy showed normal mucosa with no evidence of malignancy or polyps. Following a multidisciplinary discussion and exclusion of neoplastic disease, the elevated CEA was attributed to chronic gallbladder inflammation in the context of calculous cholecystitis, with potential contributing roles from post-bariatric nutritional deficiencies and H. pylori-associated gastritis. The patient subsequently underwent laparoscopic cholecystectomy. 

**Figure 1:**
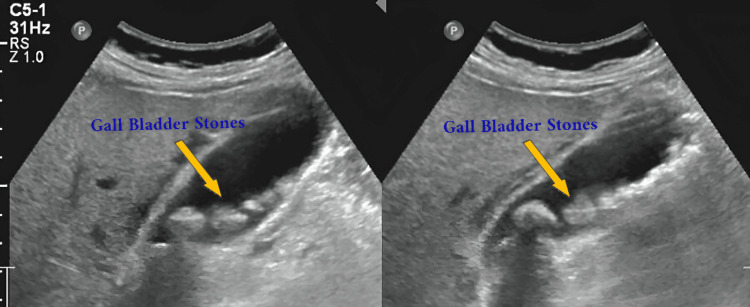
Imaging studies showing gall bladder stones (indicated by the yellow arrows)

As mentioned above, the grayscale abdominal ultrasound views demonstrated a thick-walled gallbladder with intraluminal echogenic foci with posterior acoustic shadowing, consistent with multiple gall stones (Figure 1).

The gall bladder wall appeared mildly thickened, supporting a diagnosis of chronic calcular cholecystitis. No pericholecystic fluid or biliary ductal dilation was observed. The images were obtained during the patient’s initial diagnostic workup.

At the six-week follow-up after the operation, the patient reported a significant improvement in the gastrointestinal symptoms. A third CEA measurement, performed three months after surgery and six months after her initial presentation, showed a marked decline to 2.1 ng/mL, within the normal range. This normalization supported the conclusion of a benign inflammatory cause for the prior elevation.

## Discussion

This case highlights a clinically important but often overlooked phenomenon: the elevation of CEA in benign conditions such as chronic calculous cholecystitis. While CEA remains a valuable tumor marker in selected oncology settings, particularly in the follow-up of colorectal, gastric, and pancreatic cancers, its utility is limited in asymptomatic individuals due to its low specificity [1,2]. As illustrated in this case, the indiscriminate use of CEA testing can lead to a diagnostic cascade, unnecessary anxiety, and resource-intensive investigations. CEA is a glycoprotein that functions as an oncofetal antigen, re-expressed in several malignancies. However, elevated levels may also result from non-neoplastic processes, including inflammatory bowel disease, peptic ulcer disease, cirrhosis, chronic obstructive pulmonary disease, and biliary inflammation [3-5].

In this patient, the markedly elevated CEA level initially raised concern for gastrointestinal malignancy, especially in the presence of anemia, weight loss, and a family history of colorectal cancer. However, malignancy was extensively ruled out: three repeated stool occult blood tests were negative, and cross-sectional imaging with contrast-enhanced CT showed no suspicious lesions. Both colonoscopy and upper GI endoscopy were unremarkable, except for mild gastritis and a positive H. pylori test. Further investigation failed to identify a malignant source, and the elevation was ultimately attributed to chronic gallbladder inflammation. Previous studies have shown that biliary tract diseases, particularly cholecystitis, can cause moderate to high elevations in serum CEA levels, likely due to inflammatory cytokine-mediated upregulation or impaired hepatic clearance [6]. Although mild CEA elevation (<10 ng/mL) has been reported in benign conditions, values exceeding 40 ng/mL are rare in the absence of malignancy, adding to the diagnostic complexity in this case. Additionally, the nutritional deficiencies post-bariatric surgery may further complicate the clinical picture by mimicking constitutional symptoms typically associated with malignancy, such as weight loss and fatigue. These overlapping features highlight the importance of contextual interpretation of tumor markers.

In this case, the patient’s liver function tests, including bilirubin, transaminases, and alkaline phosphatase, were within normal range, helping to exclude hepatobiliary dysfunction as a confounder. Inflammatory markers, including CRP and procalcitonin, were also unremarkable. The normalization of the patient's CEA level following laparoscopic cholecystectomy further supports the diagnosis of a benign inflammatory etiology. A follow-up test performed three months after surgery (and six months after the initial CEA elevation) showed normalization to 2.1 ng/mL. This reinforces the concept that CEA should not be used as a standalone diagnostic tool and must be interpreted in conjunction with the clinical presentation, imaging, and histopathological findings. The broader implication of this case is the cautionary tale against overuse of tumor markers in routine health screenings, especially in asymptomatic patients. Multiple clinical guidelines, including those from American Society of Clinical Oncology (ASCO), advise against CEA testing for initial cancer diagnosis or general screening due to the high risk of false positives and downstream consequences [2,7,9]. Future research should focus on identifying clinical and biochemical patterns that distinguish benign from malignant causes of elevated tumor markers. Additionally, public health policies may benefit from regulating the inclusion of tumor marker testing in commercial health check-up packages to prevent misuse.

## Conclusions

This case highlights that markedly elevated CEA levels can occur in benign conditions such as chronic calculous cholecystitis. In asymptomatic individuals, indiscriminate tumor marker testing may lead to unnecessary anxiety and extensive investigations. The normalization of CEA levels after cholecystectomy in the present case supports a non-malignant inflammatory cause. Clinicians should interpret tumor markers within the full clinical context and avoid using them for routine screening in asymptomatic patients, as emphasized by the current guidelines.

## References

[1] Duffy MJ (2013). Tumor markers in clinical practice: a review focusing on common solid cancers. Med Princ Pract.

[2] Locker GY, Hamilton S, Harris J (2006). ASCO 2006 update of recommendations for the use of tumor markers in gastrointestinal cancer. J Clin Oncol.

[3] Fletcher RH (1986). Carcinoembryonic antigen. Ann Intern Med.

[4] Gold P, Freedman SO (1965). Demonstration of tumor-specific antigens in human colonic carcinomata by immunological tolerance and absorption techniques. J Exp Med.

[5] Sturgeon CM, Duffy MJ, Stenman UH (2008). National Academy of Clinical Biochemistry laboratory medicine practice guidelines for use of tumor markers in testicular, prostate, colorectal, breast, and ovarian cancers. Clin Chem.

[6] Lurie BB, Loewenstein MS, Zamcheck N (1975). Elevated carcinoembryonic antigen levels and biliary tract obstruction. JAMA.

[7] Loewenstein MS, Zamcheck N (1978). Carcinoembryonic antigen (CEA) levels in benign gastrointestinal disease states. Cancer.

[8] Deyo RA (2002). Cascade effects of medical technology. Annu Rev Public Health.

[9] Henry NL, Hayes DF (2012). Cancer biomarkers. Mol Oncol.

[10] Davidson KW, Barry MJ, Mangione CM (2021). Screening for colorectal cancer: US Preventive Services Task Force recommendation statement. JAMA.

[11] Wolf AM, Fontham ET, Church TR (2018). Colorectal cancer screening for average-risk adults: 2018 guideline update from the American Cancer Society. CA Cancer J Clin.

